# Evaluation of Genotype MTBDR*plus* and MTBDR*sl* Assays for Rapid Detection of Drug Resistance in Extensively Drug-Resistant *Mycobacterium tuberculosis* Isolates in Pakistan

**DOI:** 10.3389/fmicb.2018.02265

**Published:** 2018-09-26

**Authors:** Hasnain Javed, Zofia Bakuła, Małgorzata Pleń, Hafiza Jawairia Hashmi, Zarfishan Tahir, Nazia Jamil, Tomasz Jagielski

**Affiliations:** ^1^Department of Microbiology and Molecular Genetics, University of the Punjab, Lahore, Pakistan; ^2^Department of Applied Microbiology, Faculty of Biology, Institute of Microbiology, University of Warsaw, Warsaw, Poland; ^3^Provincial TB Control Program, Lahore, Pakistan

**Keywords:** Genotype MTBDR*plus*, Genotype MTBDR*sl*, line probe assay, *Mycobacterium tuberculosis*, drug resistance

## Abstract

Pakistan ranks 5th among the world's highest tuberculosis (TB) burden countries alongside the 6th among countries with the highest burden of drug-resistant TB, including multi-drug resistant (MDR)-TB. Methods for rapid and reliable drug susceptibility testing (DST) are prerequisite for the prompt institution of effective anti-TB treatment. The aim of this study was to evaluate the efficiency of Genotype MTBDR*plus* and MTBDR*sl* assays for the detection of MDR and (pre-) extensively drug-resistant (XDR-TB) isolates in Pakistan. The study included 47 pre-XDR and 6 XDR-TB isolates, recovered from 53 patients from Pakistan. Conventional DST was performed using the standard 1% proportion method on the Löwenstein-Jensen medium. For molecular determination of drug resistance, GenoType MTBDR*plus* and GenoType MTBDR*sl* assays (Hain Lifescience, Germany) were used. To evaluate discrepancies between conventional and molecular DST results, mutation profiling was performed by amplifying and sequencing seven genetic loci, i.e., *katG, inhA*, and *mabA*-*inhA* promoter, *rpoB, gyrA, embB, rrs*. The sensitivity of Genotype MTBDR*plus* was 71.7% for isoniazid (INH) and 79.2% for rifampicin (RIF). Sequence analysis revealed non-synonymous mutations in 93.3 and 27.3% of isolates phenotypically resistant to INH and RIF, respectively, albeit susceptible when tested by GenoType MTBDR*plus*. GenoType MTBDR*sl* had a sensitivity of 73.6, 64.7, 20, 25, and 100% for the detection of fluoroquinolones, ethambutol, kanamycin, amikacin, and capreomycin resistance, respectively. Upon sequencing, mutations were detected in 20, 77.8%, and all isolates phenotypically resistant to aminoglycosides, ethambutol, and fluoroquinolones, respectively, yet declared as susceptible with GenoType MTBDR*sl*. Low sensitivities seriously impede the large-scale application of the Genotype MTBDR*plus* and MTBDR*sl* assays. Unless further optimized, the currently available line-probe assays should rather be auxiliary to the conventional, phenotype-based methods in the detection of MDR- and XDR-TB in Pakistan.

## Introduction

Tuberculosis (TB) remains an inglorious leader among infectious diseases in mortality, with its annual toll of 1.5 million lives worldwide (Ullah et al., 2016). Although the global TB incidence has been on a downward trend since 2000, the emergence and persistence of drug-resistant (DR) tubercle bacilli strains, particularly those multi-drug resistant (MDR), defined as resistant to at least isoniazid (INH) and rifampicin (RIF), and extensively drug-resistant (XDR), defined as MDR with additional resistance to a fluoroquinolone (FQ) and a second-line injectable drug (SLID), have substantially undermined efforts to control and eliminate the disease. According to a most recent World Health Organization (WHO) report, every seventeenth TB patient expels MDR bacilli. One in sixteen of such patients expel strains of XDR phenotype (World Health Organization, 2017). For almost two decades, Pakistan, with a population of 193 million people, continues to be on the top of the list high TB-burden countries (HBCs), in terms of total TB caseload (TB incidence rate, 268 per 100,000 population) and in terms of DR-TB, including MDR-TB (MDR-TB incidence rate, 14 per 100,000 population), globally (World Health Organization, 2017). Studies on the prevalence of DR-TB, including MDR-TB and XDR-TB in Pakistan are quite scarce and fragmentary (Ali et al., 2011, 2015; Farooqi et al., 2012; Javaid et al., 2016). Also, data collected from national surveys on DR-TB and reported to WHO have to be cautiously treated. The true prevalence of DR-TB is thought to be underestimated, mostly due to a limited number of facilities offering drug susceptibility testing (DST) and poor availability of modern and advanced technologies, allowing for fast and reliable drug resistance profiling (Domínguez et al., 2016).

Conventional DST is laborious, time consuming and requires growth of mycobacteria either on solid or liquid media (Bernard et al., 2015). Identification of the XDR phenotype is particularly protracted as it is a two-step procedure that first involves testing against first-line anti-TB drugs, and then against second-line agents, once multidrug resistance is disclosed (Abebe et al., 2011). Rapid and reliable DST results are prerequisite for the prompt implementation of effective treatment, and reducing the risk of acquired resistance (Hillemann et al., 2009). Over the last decade, diagnosing DR-TB has been greatly improved and expedited with the introduction of various molecular-based DST technologies (Thumamo et al., 2012). They all fall into two major categories that is probe-based and sequence-based methods. The former are represented by Cepheid's GeneXpert MTB/RIF assay (Cepheid, USA) with molecular beacon probes (Ioannidis et al., 2011) and Hain's MTBDR*plus* and MTBDR*sl* assays (Hain Life Sciences, Germany) with line probes (Nathavitharana et al., 2017). Whereas molecular beacon-based assays use real-time PCR, dual-labeled probes that form a quenched, stem-loop structure in native state and fluoresce upon hybridization to the target nucleotide sequence (Lawn and Nicol, 2011), line probe assays involve PCR and reverse hybridization with specific oligonucleotide probed fixed to a nitrocellulose strip in parallel lines (World Health Organization, 2008). Sequence-based methods include pyrosequencing (Molina-Moya et al., 2017), Sanger sequencing (Schleusener et al., 2017), and next-generation sequencing (Jagielski et al., 2016).

The implementation of routine sequencing to track drug- resistance profiles has already become a part of a strategy aiming at TB elimination in England, and toward similar plans in other high-income countries (Walker et al., 2017; Satta et al., 2018). However, DNA sequencing-based approaches remains prohibitevely expensive and complex for routine use in low- and middle-income countries, especially those heavily burdened with TB (Votintseva et al., 2017).

In December 2010 WHO approved the GeneXpert MTB/RIF test for detection of DR strains in high-burden, resource-limited countries (World Health Organization, 2013). An important drawback of the system is that it detects resistance to RIF but not to other anti-TB drugs such as INH, FQs, and SLIDs. Furthermore, whereas PCR inhibition or unsuccessful DNA extraction may increase the chances of false-negative results, false positive results might occur in previously treated patients and having mixed TB/non-tuberculous mycobacteria (NTM) infection (Liu et al., 2017).

The Hain probe assays use reverse hybridization technology to detect mutations associated with resistance of tubercle bacilli to both first- and second-line anti-TB drugs. MTBDR*plus* allows for identification of INH and RIF resistance by disclosing mutations in the *katG, inhA*, and *rpoB* genes, while MTBDR*sl* detects resistance to FQs, ethambutol (EMB), aminoglycosides (kanamycin, KAN; amikacin, AMK; viomycin, VIO), and cyclic peptide (capreomycin, CAP) by finding mutations in three different loci, that is *gyrA, embB*, and *rrs*, respectively (Bai et al., 2016).

Based on two recent meta-analyses, including over 50 studies, the overall sensitivities of the two Genotype assays varied widely from 96% for RIF to 44% for KAN, while the specificities ranged from 99% for INH, RIF, AMK, and KAN to 79% for EMB (Feng et al., 2013; Bai et al., 2016). Still, conventional, culture-based DST is considered a gold standard in mycobacteriology.

The aim of the study was to evaluate the efficiency of Genotype MTBDR*plus* and MTBDR*sl* assays in the detection of drug resistance in the context of conventional DST profiling and results of PCR-sequencing targeted at selected drug resistance-associated loci.

## Materials and methods

### Isolates

The study sample was selected out of a pool of 3056 *Mycobacterium tuberculosis* isolates, recovered from as many patients, originally diagnosed as having pulmonary TB and originated from seven major cities of Punjab province of Pakistan from January 2013 to June 2015. MDR-TB, based on the conventional DST results, was initially identified in 362 of the cases (Figure S1). These cases were further reviewed to select those where (pre-)XDR-TB was confirmed. Thereby, the final study group of 53 patients was achieved (Table 1).

**Table 1:** Socio-demographic and clinical characteristics of patients under the study.

**Category^a^**		**No. of patients (*n* = 53)**	**Drug susceptibility status**		***P***
			**pre-XDR**	**XDR**	
**Age**	15–29	28 (52.8%)	24 (45.3%)	4 (7.5%)	0.94
	30–44	15 (28.3%)	13 (24.5%)	2 (3.8%)	
	>45	10 (18.9%)	9 (17%)	1 (1.9%)	
**Sex**	Male	27 (50.9%)	24 (45.3%)	3 (5.7%)	0.645
	Female	26 (49.1%)	22 (41.5%)	4 (7.5%)	
**Area**	Urban	33 (62.3%)	31 (58.5%)	2 (3.7%)	0.048^*^
	Rural	20 (37.7%)	15 (28.3%)	5 (9.4%)	
**Treatment history**	New case	3 (5.7%)	3 (5.7%)	0 (0%)	NA^b^
	Retreatment	50 (94.4%)	43 (81.1%)	7 (13.2%)	
	Default	22 (44%)	17 (34%)	5 (10%)	0.28
	Relapse (*n* = 50)^a^	15 (30%)	14 (28%)	1 (2%)	
	Failure	13 (26%)	12 (24%)	1 (2%)	

a *Data were available for 53 patients, otherwise indicated*.

An asterisk indicates a difference statistically significant.

b *Non-applicable. Since there were no new XDR-TB cases (n = 0), the statistical test could not be performed*.

pre-XDR phenotype was defined as MDR with either resistance to ofloxacin (OFX) or any of the SLIDs (i.e., AMK, KAN or CAP). XDR-TB was defined as MDR-TB with additional resistance to OFX and one of the SLIDs.

Primary isolation, culturing, and species identification were performed with standard mycobacteriological methods in Provincial TB Reference Laboratory, Institute of Public Health, Lahore. Briefly, clinical samples were decontaminated with 1% N-acetyl-L-cysteine/NaOH and centrifuged. Each sample was then inoculated onto Löwenstein-Jensen and MGIT (Becton Dickinson, Franklin Lakes, NJ, USA) media. Identification was performed using the BACTEC NAP TB Differentiation Test Kit (Becton Dickinson, USA), growth in para-nitrobenzoic acid–containing media, nitrate reduction, and niacin accumulation (Koneman et al., 1983).

This study was carried out in accordance with the recommendations of ethical policy of the University of Punjab.

### Drug susceptibility testing

Conventional DST was performed using the standard 1% proportion method on the Löwenstein-Jensen (L-J) medium, with the *M. tuberculosis* H37Rv strain as a quality control, following the WHO recommendations (Van Embden et al., 1993). The critical concentrations for specific drugs were as follows: INH, 0.2 mg/L; RIF, 40 mg/L; EMB, 2 mg/L; STR, 4 mg/L; KAN, 30 mg/L; AMK, 30 mg/L; CAP, 40 mg/L; and OFX, 4 mg/L.

For molecular determination of drug resistance, GenoType MTBDR*plus* and GenoType MTBDR*sl* assays (Hain Lifescience, Germany) were used. The principle of these tests was essentially described elsewhere (Anek-vorapong et al., 2010). Both assays were performed and interpreted in accordance with the manufacturer's instructions. A test result was considered valid, only if all control bands appeared correctly. An isolate was declared resistant if at least one wild type probe was absent or if any mutant probe was present. If all wild type probes were present and all mutant probes were absent, an isolate was recognized as susceptible.

### DNA extraction

Genomic DNA from *M. tuberculosis* isolates, grown on L-J medium, was extracted using the cetyl-trimethyl ammonium bromide (CTAB) method, as described previously (Jagielski et al., 2015).

### PCR and sequencing

To evaluate discrepancies between conventional and molecular (GenoType MTBDR*plus* and MTBDR*sl*) DST results, selected fragments of seven genetic loci, containing the hot spots for mutations associated with resistance of tubercle bacilli to INH (*katG, inhA*, and *mabA*-*inhA* promoter), RIF (*rpoB*), EMB (*embB*), aminoglycosides (*rrs*), and FQs (*gyrA*) were PCR-amplified and sequenced. The oligonucleotide primers used for PCR are described in Table S1. Amplification reactions were set up and performed according to the manufacturer's instructions (TopTaq DNA polymerase, Qiagen, Germany). Purified PCR amplicons (Clean-up kit, A&A Biotechnology, Poland) were sequenced in both directions using the same primers as for PCR amplification. Mutations were detected using Clone Manager software (v. 8.0, Scientific & Educational Software, USA) by comparing the obtained sequences with the *M. tuberculosis* reference strain H37Rv sequences of respective loci, deposited in the GenBank database (National Center for Biotechnology Information; http://www.ncbi.nlm.nih.gov/). An isolate was declared resistant if at least one non-synonymous mutation was detected in a resistance-associated locus.

Nucleotide bases and codon numbers were reported using either *M. tuberculosis* (*katG, inhA, mabA*-*inhA, embB, rrs, gyrA*) or *Escherichia coli* (*rpoB*) numbering system.

### Nucelotide accession numbers

The sequences with detected mutations were deposited in GenBank (National Center for Biotechnology Information; http://www.ncbi.nlm.nih.gov/) under the following accession numbers: MF145294-MF145302, MF145309, MF145313 for the *rpoB* gene, MF145303-MF145308, MF145310-MF145312, MF145314-MF145316 for the *katG* gene, MF145317-MF145331 for the *gyrA* gene, MF145332-MF145339 for *rrs* gene, and MF145340- MF145357 for the *embB* gene.

### Statistical analysis

Data were analyzed with the IBM SPSS software (version 22.0, USA). To analyze differences between categorical variables, χ^2^-test was used. A value of *P* < 0.05 was considered statistically significant.

### Diagnostic performance

To assess the diagnostic performance of the line-probe assays, sensitivities and positive predictive values (PPV) were calculated for all drugs covered by the GenoType MTBDR*plus* and MTBDR*sl* assays. Since the study sample included no INH-, RIF-, FQ-susceptible isolates, specificities and negative predictive values (NPV) were calculated only for KAN, AMK, CAP, and EMB of GenoType MTBDR*sl*.

## Results

### Conventional DST

All (53/53) isolates tested were resistant to INH, RIF (MDR) and OFX. Fifty-one (51/53; 96.2%) isolates were resistant to EMB, 39 (39/53; 73.6%) to STR, five (5/53; 9.4%) to KAN, and four (4/53; 7.5%) to AMK. Only one (1/53; 1.9%) isolate was CAP-resistant (Table 2). The most common drug resistance pattern was INH+RIF+OFX+EMB+STR (29/53, 54.7%) followed by INH+RIF+OFX+EMB (12/53, 22.6%). Forty-seven (47/53; 88.7%) isolates were resistant to OFX and not to any other SLIDs, thus meeting the definition of pre-XDR-TB. Six (6/53; 11.3%) isolates were categorized as XDR-TB isolates with additional (to INH+RIF+OFX) resistance to AMK and KAN (2/53; 3.8%), KAN (2/53; 3.8%), AMK (1/53; 1.9%), and AMK, KAN and CAP (1/53; 1.9%) (Table 2).

**Table 2:** Drug susceptibility profiles detected by conventional DST, GenoType MTBDR*plus*, GenoType MTBDR*sl* (LPA), and sequence analysis (SEQ).

**Strain ID**	**INH**			**RIF**			**FLQ**			**KAN**			**AMK**			**VIO**			**CAP**			**EMB**		
	**DST**	**LPA**	**SEQ**	**DST**	**LPA**	**SEQ**	**DST**	**LPA**	**SEQ**	**DST**	**LPA**	**SEQ**	**DST**	**LPA**	**SEQ**	**DST**	**LPA**	**SEQ**	**DST**	**LPA**	**SEQ**	**DST**	**LPA**	**SEQ**
A2	R	R	X	R	R	X	R	R	X	S	S	X	S	S	X	X	S	X	S	S	X	R	R	X
A5	R	R	X	R	R	X	R	R	X	R	S	S	R	S	S	X	S	X	X	S	X	R	R	X
A6	R	R	X	R	R	X	R	R	X	S	R	R	S	R	R	X	R	X	X	R	X	R	R	X
A7	R	R	X	R	R	X	R	R	X	S	S	X	S	S	X	X	S	X	X	S	X	R	R	X
A8	R	R	X	R	R	X	R	S	R	S	S	X	S	S	X	X	S	X	X	S	X	R	R	X
A14	R	R	X	R	R	X	R	S	R	S	S	X	S	S	X	X	S	X	S	S	X	R	R	X
A16	R	S	R	R	S	S	R	R	X	S	S	X	X	S	X	X	S	X	X	S	X	R	R	X
A17	R	R	X	R	R	X	R	R	X	S	S	X	S	S	X	X	S	X	S	S	X	R	R	X
A18	R	R	X	R	R	X	R	R	X	S	S	X	S	S	X	X	S	X	S	S	X	R	S	R
A19	R	R	X	R	R	X	R	S	R	S	S	X	S	S	X	X	S	X	S	S	X	R	R	X
A20	R	R	X	R	R	X	R	S	R	S	R	S	X	R	X	X	S	X	X	R	X	R	R	X
A21	R	R	X	R	R	X	R	S	R	S	R	S	X	R	X	X	S	X	X	R	X	R	R	X
A22	R	R	X	R	R	X	R	S	R	R	S	S	R	S	S	X	S	X	X	S	X	R	R	X
A23	R	R	X	R	R	X	R	S	R	S	S	X	S	S	X	X	S	X	S	S	X	R	R	X
A24	R	R	X	R	R	X	R	R	X	S	S	X	R	S	S	X	S	X	X	S	X	R	R	X
A26	R	R	X	R	R	X	R	R	X	S	S	X	S	S	X	X	S	X	S	S	X	R	R	X
A31	R	R	X	R	R	X	R	R	X	S	S	X	S	S	X	X	S	X	S	S	X	R	R	X
A33	R	R	X	R	R	X	R	R	X	S	S	X	S	S	X	X	S	X	S	S	X	R	R	X
A35	R	R	X	R	R	X	R	S	R	S	S	X	S	S	X	X	S	X	S	S	X	R	R	X
A36	R	R	X	R	S	R	R	R	X	S	S	X	S	S	X	X	S	X	S	S	X	S	R	R
A37	R	S	R	R	S	S	R	R	X	S	S	X	S	S	X	X	S	X	S	S	X	R	R	X
2.A12	R	R	X	R	R	X	R	S	R	S	S	X	S	S	X	X	S	X	S	S	X	R	R	X
A49	R	S	R	R	S	R	R	S	R	S	S	X	S	S	X	X	S	X	S	S	X	R	R	X
A68	R	R	X	R	R	X	R	R	X	S	S	X	X	S	X	X	S	X	X	S	X	R	S	S
A162	R	S	R	R	S	S	R	S	R	S	S	X	S	S	X	X	S	X	S	S	X	R	S	R
A163	R	R	X	R	R	X	R	R	X	S	S	X	S	S	X	X	S	X	S	S	X	R	S	R
A164	R	R	X	R	R	X	R	R	X	R	S	S	S	S	X	X	S	X	S	S	X	S	S	X
A165	R	R	X	R	R	X	R	R	X	S	R	S	S	R	S	X	S	X	S	R	S	R	R	X
A172	R	R	X	R	R	X	R	R	X	S	S	X	S	S	X	X	S	X	S	S	X	R	S	R
A175	R	S	R	R	R	X	R	R	X	S	S	X	S	S	X	X	S	X	S	S	X	R	S	R
A186	R	R	X	R	S	S	R	R	X	S	S	X	S	S	X	X	S	X	S	S	X	R	S	R
A194	R	S	S	R	R	X	R	R	X	S	S	X	S	S	X	X	S	X	S	S	X	R	R	X
A202	R	S	R	R	S	S	R	S	R	S	S	X	S	S	X	X	S	X	S	S	X	R	S	S
A254	R	R	X	R	R	X	R	R	X	S	S	X	S	S	X	X	S	X	S	S	X	R	R	X
A315	R	S	R	R	R	X	R	R	X	S	S	X	S	S	X	X	S	X	S	S	X	R	R	X
A321	R	R	X	R	R	X	R	R	X	R	R	X	R	R	X	X	S	X	R	R	X	R	R	X
A323	R	R	X	R	S	S	R	R	X	S	S	X	S	S	X	X	S	X	S	S	X	R	R	X
A331	R	R	X	R	R	X	R	R	X	S	S	X	S	S	X	X	S	X	X	S	X	R	R	X
A332	R	R	X	R	R	X	R	R	X	S	S	X	S	S	X	X	S	X	S	S	X	R	R	X
A344	R	S	R	R	R	X	R	R	X	S	S	X	S	S	X	X	S	X	S	S	X	R	S	S
A345	R	R	X	R	S	R	R	R	X	S	R	R	S	R	R	X	R	X	S	R	R	R	R	X
A346	R	S	R	R	R	X	R	S	R	S	S	X	S	S	X	X	S	X	S	S	X	R	S	R
A350	R	S	R	R	R	X	R	R	X	S	S	X	S	S	X	X	S	X	S	S	X	R	S	R
A368	R	S	R	R	R	X	R	R	X	S	R	S	S	R	S	X	S	X	S	R	S	R	R	X
A370	R	R	X	R	R	X	R	R	X	S	S	X	S	S	X	X	S	X	S	S	X	R	S	R
A373	R	S	R	R	R	X	R	R	X	S	S	X	S	S	X	X	S	X	S	S	X	R	S	S
A378	R	R	X	R	R	X	R	R	X	S	S	X	S	S	X	X	S	X	S	S	X	R	S	R
A381	R	S	R	R	S	S	R	S	R	S	R	S	S	R	S	X	S	X	S	R	S	R	S	R
A382	R	S	R	R	R	X	R	R	X	S	S	X	S	S	X	X	S	X	S	S	X	R	S	R
A398	R	R	X	R	R	X	R	R	X	S	S	X	S	S	X	X	S	X	S	S	X	R	R	X
A408	R	R	X	R	S	S	R	R	X	R	S	S	S	S	X	X	S	X	S	S	X	R	R	X
A410	R	R	X	R	R	X	R	R	X	S	S	X	S	S	X	X	S	X	S	S	X	R	S	R
A422	R	R	X	R	R	X	R	R	X	S	S	X	S	S	X	X	S	X	S	S	X	R	S	R
Total R	53	38	14	53	42	3	53	39	14	5	8	2	4	8	2	–	2	–	1	8	1	51	34	15
Total S	–	15	1	–	11	8	–	14	–	48	45	9	45	45	6	–	51	–	41	45	3	2	19	4

*R, resistant; S, susceptible; X, not analyzed*.

### Performance of genotype MTBDR*plus*

**Figure 1:**
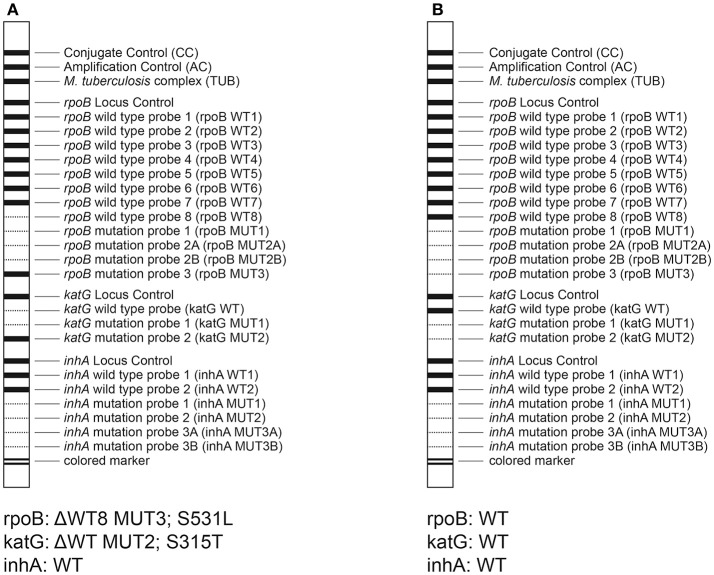
Representative patterns obtained by the MTBDR*plus* assay. **(A)** isolate 2A12, RIF^r^, and INH^r^; **(B)** isolate A16, RIF^s^, and INH^s^.

The results of DST with the GenoType MTBDR*plus* assay are shown in Table 2, while representative patterns obtained with the assay are depicted in Figure 1.

Detailed patterns of drug resistance are given in Table S2. Resistance to INH was detected in 38 (38/53; 71.7%) isolates. Of these, all but one (37/53; 69.8%) had S315T (MUT1) alteration in the *katG* gene along with a WT band present (21/53; 39.6%) or absent ΔWT (16/53; 30.2%). Four (4/53; 7.5%) isolates had mutations in the *inhA* promoter. Single polymorphism C-15T occurred in three (3/53; 5.6%) isolates (MUT1+ΔWT1, 2/53, 3.8%; MUT1 coupled with WT bands, 1/53, 1.9%). One (1/53; 1.9%) isolate harbored two substitutions, i.e., C-15T and T-8C (MUT1 and MUT3A, with all WT bands present). All but one isolates with mutations in the *inhA* promoter had their *katG* alleles unreactive to mutant probes.

GenoType MTBDR*plus* identified 42 (42/53; 79.2%) isolates as RIF-resistant. The most prevalent mutation pattern was S531L (MUT3) found in 34 (34/53; 64.1%) isolates, coupled with either ΔWT8 (S531L; 32/53; 60.4%), ΔWT3ΔWT4 (D516Y/Δ515; 1/53; 1.9%) or all WT bands (1/53; 1.9%). Five (5/53; 9.4%) and two (2/53; 3.8%) isolates carried mutations in codons 515–516 (ΔWT3ΔWT4, D516Y/Δ515, 3/53, 5.7%; MUT1+ΔWT3ΔWT4, D516V+D516Y/Δ515, 1/53, 1.9%; MUT1+ΔWT7, D516V+H526R/P/W/N/L/S/C, 1/53, 1.9%) and 526 (ΔWT7, H526R/P/W/N/L/S/C), respectively. One (1/53; 1.9%) isolate had S522L/Q (ΔWT5ΔWT6) and L533P/S531Q/W (ΔWT8) mutations.

Concordance between DST results obtained with the MTBDR*plus* assay and conventional methods is shown in Table 3. The sensitivity of the MTBDR*plus* assay was calculated at 79.2% and 71.7% for the detection of RIF and INH resistance, respectively.

**Table 3:** GenoType MTBDR*plus* and GenoType MTBDR*sl* assays characteristics.

**Category**	**GenoType MTBDR*****plus***		**GenoType MTBDR*****sl***				
	**INH**	**RIF**	**FQ**	**KAN**	**AMK**	**CAP**	**EMB**
Sensitivity (%)	71.7	79.2	73.6	20	25	100	64.7
Specificity (%)	NA	NA	NA	85.4	88.9	90.2	50
PPV (%)	100	100	100	12.5	16.7	20	97
NPV (%)	NA	NA	NA	91.1	93	100	5.3
Agreement (%)	71.7	79.2	73.6	79.2	83.7	90.5	64.2

*PPV, positive predictive value; NPV, negative predictive value; NA, non-applicable, none of the isolates had a negative result with upon conventional DST method, thus this measure could not be calculated*.

### Performance of genotype MTBDR*sl*

The results of DST with the GenoType MTBDR*sl* are shown in Table 2. Detailed patterns of drug resistance are given in Table S2.

Resistance to FQ was detected in 39 (39/53; 73.6%) isolates. Mutations A90V (MUT1+ΔWT2) and D94G (MUT3C+ΔWT3) in the *gyrA* gene occurred with equal frequencies, that is in 13 (13/53; 24.5%) isolates, each. The S91P (MUT2+ΔWT2 or MUT2 and all WT bands), D94N/Y (MUT3B+ΔWT3 or MUT3B and all WT bands), D94H (MUT3D+ΔWT3), and D94A (MUT3A+ΔWT3) alterations were detected in 3 (3/53; 5.6%), 3 (3/53; 5.6%), 2 (2/53; 3.8%), and 2 (2/53; 3.8%) isolates, respectively. Three (3/53; 5.6%) isolates carried a double amino acid change; two isolates had A90V and D94G (MUT1+MUT3C, all WT bands) mutations, whereas one had D94N/Y and D94G (MUT3B+MUT3C+ΔWT3) mutations.

Resistance to EMB was detected in 33 (33/53; 63.3%) isolates. The most prevalent mutation was M306I, found in 18 (18/33; 54.5%) isolates, of which 9 (9/33; 27.3%) had MUT1A+ΔWT and another 9 had ΔWT banding patterns. Fifteen (15/33; 45.5%) isolates harbored M306V (MUT1B+ΔWT) alteration. Of two isolates, identified as EMB-susceptible, upon conventional DST, one was recognized as EMB-resistant with the assay (ΔWT pattern).

Out of six isolates resistant to SLIDs upon conventional DST, only one (1/53; 1.9%) was found resistant (KAN+AMK+CAP) with the MTBDR*sl* assay. This isolate carried a A1401G (MUT1+ΔWT1) mutation. Seven (7/53; 13.2%) isolates were designated as KAN-, AMK- and/or CAP-resistant, despite being susceptible to those drugs upon culture-based DST. Mutations A1401G (MUT1+ΔWT1 or MUT1 and all WT bands) or G1484T (MUT2+ΔWT2) were found in five (5/7; 71.4%) and two (2/7; 28.6%) of these isolates, respectively.

Overall, GenoType MTBDR*sl* had sensitivities of 73.6, 64.7, 20, 25, and 100% for the detection of FQ, EMB, KAN, AMK, and CAP resistance. Whereas the specificities ranged from 90.2% for CAP, 88.9% for AMK, 85.4% for KAN to 50% for EMB (Table 2).

### Mutation profiling

Of the 11 (11/53; 20.8%) isolates declared as RIF-resistant but RIF-susceptible with the conventional and GenoType MTBDR*plus* assay, respectively, three (3/11; 27.3%) harbored non-synonymous alterations S531L, S531W, and I572F upon *rpoB* gene sequencing. Another seven (7/11; 63.6%) isolates had silent mutations, and one (1/11; 9.1%) isolate had a WT *rpoB* sequence (Table 2, Table S2).

Among 15 (15/53; 28.3%) isolates in which MTBDR*plus* assay failed to detect INH resistance, 14 (14/15; 93.3%) had amino acid replacements in the *katG* gene evidenced by sequencing. The mutation profiles were as follows: R463L (10/15; 66.7%), R463L and S315T (1/15; 6.7%), R463L and S315N (1/15; 6.7%), R463L and P232R (1/15; 6.7%), or R463L and D189Y (1/15; 6.7%). None of the analyzed isolates carried polymorphisms in the *inhA* promoter and structural gene.

Fourteen (14/53; 26.4%) isolates designated as FQ-resistant with conventional DST, showed FQ susceptibility with the GenoType MTBDR*sl* assay. Sequence analysis of the *gyrA* gene revealed that all these isolates carried a S95T substitution, which in case of five (5/14; 35.7%) isolates co-occurred with either A90V, S91P, D94A, D94G, or D94Y polymorphism.

Among 18 isolates for which MTBDR*sl* assay failed to confirm phenotype-based resistance to EMB, 14 (14/18; 77.8%) had mutations in the *embB* gene, as shown upon sequence analysis. These mutations translated into the following amino acid alterations: Q497P (2/18; 11.1%), Q497K (2/18; 11.1%), G406D (2/18; 11.1%), Q497R (1/18; 5.5%), G406A (1/18; 5.5%), G406S (1/18; 5.5%), D328I (1/18; 5.5%), D328Y (1/18; 5.5%), M306V (1/18; 5.5%), I284V+C549W (1/18; 5.5%), and D328Y+E378A (1/18; 5.5%). One isolate declared as EMB-susceptible upon conventional DST and as EMB-resistant with GenoType MTBDR*sl*, carried M306L mutation.

Among five isolates phenotypically resistant to SLIDs yet missed by the GenoType MTBDR*sl* assay, only one (1/5; 20%) carried a mutation (C517T) in the *rrs* gene. Two (2/7; 28.6%) out of seven isolates identified as resistant to SLIDs with the GenoType MTBDR*sl* assay but susceptible with phenotypic DST, carried a single mutation (i.e., G482A or C517T) in their *rrs* genes.

## Discussion

Genotype MTBDR*plus* and MTBDR*sl* have become increasingly popular in mycobacteriology laboratories serving as fast molecular assays for detection of drug resistance. However, studies exploring the diagnostic performance of these two assays in HBCs are seriously lacking. This work evaluates the usefulness of Genotype MTBDR*plus* and MTBDR*sl* for the detection of drug resistance in *M. tuberculosis* isolates in Pakistan. It is also the first study from Pakistan investigating drug resistance profiles of tubercle bacilli (MDR-TB isolates) with a three-pronged approach, that is conventional, culture-based method, line-probe, hybridization assays, and PCR sequencing.

The sensitivities of the Genotype MTBDR*plus* assay evidenced here were lower than those published previously from Pakistan (71.7 vs. 76.3 and 88.8% for INH; 79.2 vs. 90.2 and 92.5% for RIF) (Farooqi et al., 2012; Javaid et al., 2016) and other countries (88–94% for INH; 95–97% for RIF) (Bai et al., 2016).

Most of the isolates had S315T and S531L mutations in the *katG* and *rpoB* genes, respectively. This is in line with what was observed among MDR isolates by using not only line-probe assays (69.8 vs. 55.9–90.6% for S315T *katG;* 64.1 vs. 41.4–67.2% for S531L *rpoB*) (Farooqi et al., 2012; Shubladze et al., 2013; Sharma et al., 2014; Javaid et al., 2016; Spinato et al., 2016) but also sequencing strategy (66–76% for S315T *katG;* 46.3–66% for S531L *rpoB*) (Jou et al., 2005; Ali et al., 2011; Makadia et al., 2012; Jagielski et al., 2015; Unissa et al., 2015). Both these mutations are thought to be low fitness cost mutations, with no adverse effect on transmission capacity (Gagneux, 2009).

Among isolates in which Genotype MTBDR*plus* assay failed to detect INH resistance, all but one had a missense mutation in codon 463 of the *katG* gene, as evidenced by PCR-sequencing. This codon is not covered by any of the probe of the Genotype MTBDR*plus* assay. Mutations at codon 463 of the *katG* gene have been identified in both INH-resistant (29–59.2%) and INH-susceptible (32–63%) strains, suggesting that they not directly associated with INH resistance (Van Doorn et al., 2001; Arjomandzadegan et al., 2011; Torres et al., 2015). One isolate carried a *katG* S315N mutation, which had previously been correlated with INH-resistant phenotype (Wei et al., 2003). Since the Genotype MTBDR*plus* test strip has no probe specific for this mutation, it cannot be detected with the assay. Still, almost a fourth (23.8%) of INH-resistant isolates can harbor this mutation (Jin et al., 2012). Apart from codons 315 and 463, mutations in the *katG* gene were identified at two other codons i.e., 189 and 232. Only mutation in the latter codon (P232R) had previously been described in one INH-resistant isolate by (Greif et al., 2012), with a frequency of 2.2% among INH-resistant isolates. The precise role of these mutations in the development of INH resistance needs further investigation.

In case of RIF, three out of 11 isolates falsely designated as RIF-susceptible with GenoType MTBDR*plus* had missense mutations in their *rpoB* gene sequences i.e., S531L, S531W (in regions covered with the assay), and I572F (region not covered with the assay). I572F mutation had previously been linked with RIF resistance (Siu et al., 2011).

The sensitivities of the Genotype MTBDR*sl* assay were usually lower than those reported previously, falling in the ranges of 84.5–100% (vs. 73.6% in our study) for FQ, 65.2–70.9% (vs. 64.7%) for EMB, 39.6–89.2% (vs. 20%) for KAN, 77.7–93.8% (vs. 25%) for AMK, and 77.2–91.4% (vs. 100%) for CAP (Feng et al., 2013; Mao et al., 2015; Gardee et al., 2017). When compared with earlier studies from Pakistan, the sensitivities of Genotype MTBDR*sl* from this study were lower for AMK (56.6 vs. 25%) and EMB (81.8 vs. 64.7%), albeit similar for FQ (72.9 vs. 73.6%).

The most prevalent mutations involved in FQ, EMB, and SLID resistance were D94G (*gyrA*), M306I (*embB*), and A1401G (*rrs*), respectively, with their frequencies ranging from 20 to 35%. This is consistent with previous observations, where mutations D94G and M306I accounted for 32% of ciprofloxacin (CIP)-resistant and 35% of EMB-resistant isolates from Pakistan (Ali et al., 2011, 2015) and 21–32% of FQ-resistant and 22–68% of EMB-resistant isolates from other geographical locales (Bakuła et al., 2013; Avalos et al., 2015; Brossier et al., 2015). The prevalence of A1401G mutants among SLID-resistant isolates from Pakistan was within ranges of 22.2–78.4% (AMK), 22.2–78.4% (KAN), and 20–78.6% (CAP) (Ali et al., 2011, 2015). Globally, the cumulative frequency of that mutation (A1401G), based on a recent meta-analytical study, was 78, 76, and 56% among AMK-, CAP-, and KAN-resistant isolates (Georghiou et al., 2012).

All fourteen isolates falsely designated as FQ-susceptible upon GenoType MTBDR*sl* carried a S95T *gyrA* substitution, which is a natural polymorphism, not associated with FQ resistance (Bakuła et al., 2016). Five isolates carried *gyrA* alterations missed with Genotype MTBDR*sl* (i.e., A90V, S91P, D94A, D94G, or D94Y). According to the literature, they have all been associated with FQ resistance.

Fourteen out of 18 isolates in which Genotype MTBDR*sl* failed to detect EMB resistance had a change in the *embB* gene, as revealed by sequence analysis. The most frequent were amino acid changes in codons 497 and 406. Mutations at both these codons were shown to increase resistance to EMB (Safi et al., 2010). Additionally, mutations in five other codons of the *embB* gene were identified and four of them (except in codon 284) have been described as conferring resistance to EMB (Ali et al., 2015).

Among five isolates phenotypically resistant to SLIDs but missed by GenoType MTBDR*sl*, only one, with resistance to KAN, carried a mutation (C517T) in the *rrs* gene, localized in highly mutable region known as the 530 loop (Jagielski et al., 2014). Mutations in this region are associated with resistance to STR but not to other aminoglycosides (Bakuła et al., 2016).

According to this study, both GenoType MTBDR*plus* and GenoType MTBDR*sl*, assays display an important level of inconsistency with conventional DST. When compared to the latter, the line-probe assays were unable to detect 20.7, 28.3, 26.4, 35.3, 80, and 75% of isolates resistant to RIF, INH, FQ, EMB, KAN, and AMK, respectively. These false negative results may be explained by two reasons. First is that mutations not covered by the probes might be more prevalent in Pakistan compared to other geographical locales. Inclusion of probes specific for mutations at two *embB* codons (Q497 and G406) would considerably increase the sensitivity of the MTBDR*sl* assay to detect EMB-resistant isolates from our sample (i.e., from 64.7 to 82.3%). Also, addition of other probes specific for mutations conferring resistance to INH (e.g., S315N, KatG) or RMP (e.g., I572F, RpoB) would improve the sensitivity of the MTBDR*plus* by approximately 2%, each (71.7 vs. 73.6% for INH; 79.2 vs. 81.1% for RMP). Poor outcomes of the GenoType MTBDR*plus* and MTBDR*sl* assays due to the lack of probes specific for certain mutations were described in previous studies (Huang et al., 2011; Jin et al., 2012; Maschmann et al., 2013). Second are possible technical errors in phenotypic and molecular assays. Factors such as handling procedures, incubation conditions, and end-point interpretation may influence the outcome of conventional DST (Schön et al., 2017). Whereas, losses on DNA extraction or inhibitors of amplification present in the specimens may affect the efficiency of line probe assays (Mäkinen et al., 2002; Padilla et al., 2004). In this study they missed 10 mutations detected by sequencing of the *katG* (S315T, one isolate, 1.9%), *rpoB* (S531L or S531W, two isolates, 3.8%), *gyrA* (A90V, S91P, D94A, D94G or D94Y, five isolates, 9.4%), and *embB* (M306V, one isolate, 1.9%) genes. Third, drug resistance may originate from mutations at other genes than those included in the probe-line assays (e.g., in *ahpC, kasA*, and *ndh* for INH) (Ferro et al., 2013) and this may also result in false negative results.

Finally, false positive results were noted for 50, 11.1, 14.5, and 9.7% of isolates declared as susceptible to EMB, AMK, KAN, and CAP, respectively, with conventional DST. This can be explained by probe mispriming (due to sample contamination or suboptimal procedure conditions) or the presence of synonymous mutations (Ajileye et al., 2017).

To conclude, for detecting drug resistance in TB cases, especially in high TB incidence countries, such as Pakistan, molecular approaches should still be a complement rather than a replacement to conventional DST. The knowledge on frequencies of drug-resistance conferring mutations in clinically and geographically diverse settings, should guide the inclusion of new specific probes in the test strips in the line-probe assays, such as Genotype MTBDR.

## Author contributions

HJ executed the strain isolation and culturing, analyzed and interpreted the data, and wrote the manuscript. ZB analyzed and interpreted the data, and wrote the manuscript. MP executed the Genotype MTBDRplus and MTBDRsl analysis, and the sequence analysis. HH performed the species-level identification and drug susceptibility testing. ZT executed the administrative, the technical, and the material support. NJ critically revised the manuscript. TJ designed the study and supervised and wrote and critically revised the manuscript.

### Conflict of interest statement

The authors declare that the research was conducted in the absence of any commercial or financial relationships that could be construed as a potential conflict of interest.
