# Supplementary material for: Evaluation of Genotype MTBDRplus and MTBDRsl Assays for Rapid Detection of Drug Resistance in Extensively Drug-Resistant Mycobacterium tuberculosis Isolates in Pakistan
Source: Front Microbiol. 2018 Sep 26;9:2265. doi: 10.3389/fmicb.2018.02265 (PMC6169422; doi:10.3389/fmicb.2018.02265)
Supplement: Supplementary file 1 [file Data_Sheet_1.docx]

**SUPPLEMENTARY MATERIAL**

**Evaluation of Genotype MTBDR*plus* and MTBDR*sl* for rapid detection of drug resistance in extensively drug-resistant *Mycobacterium tuberculosis* isolates in Pakistan**

Hasnain Javed^a^, Zofia Bakuła^b^, Małgorzata Pleń^b^, Hafiza Jawairia Hashmi^a^, Zarfishan Tahir^c^, Nazia Jamil^a^, Tomasz Jagielski^b#^

^a^Department of Microbiology and Molecular Genetics, University of the Punjab, Lahore, Pakistan

^b^Department of Applied Microbiology, Institute of Microbiology, Faculty of Biology, University of Warsaw, Poland

^c^Provincial TB Control Program, Lahore

#Address correspondence to: Tomasz Jagielski, PhD, Department of Applied Microbiology, Institute of Microbiology, Faculty of Biology, University of Warsaw, I. Miecznikowa 1, 02-096 Warsaw, Poland. Phone: +48 (0) 22 55 41 431; Fax: +48 (0) 22 55 41 402, E‑mail: [t.jagielski@biol.uw.edu.pl](mailto:t.jagielski@biol.uw.edu.pl)

**TABLE S1. Oligonucleotide primers used for amplification and sequencing of the analyzed genes.**

| Locus | Primer | | Annealing temperature (°C) | Amplicon size (bp) |
| --- | --- | --- | --- | --- |
|  | Designation | Nucleotide sequence (5′→3′) |  |  |
| *rpoB* | Fw: rpoBBF^a^ | CCTCTAAGGGCTCTCGTTGG | 55 | 1673 |
|  | Rev: rpoBBR^a^ | GATCGGCGAATTGGCCTGTG |  |  |
| *katG* | Fw: katG119F^a^ | CCAACCGGCTCAATCTGAAG | 55 | 1346 |
|  | Rev: katG1464R^a^ | CTTGTCGCTACCACGGAACG |  |  |
| *inhA* | Fw: inhAF^a^ | AACACAAGGACGCACATGAC | 55 | 810 |
|  | Rev: inhAR^a^ | GCATGCGTTCTAGAGCAATTGG |  |  |
| *inhA-mabA* | Fw: pro1^a^ | TCAATACACCCGCAGCCA | 55 | 491 |
|  | Rev: pro2^a^ | GTCATCCGCATGAGGAAT |  |  |
| *rrs* | Fw: rrsF^a^ | CCACACTGGGACTGAGATAC | 54 | 783 |
|  | Rev: rrsR^a^ | TGCGGGACTTAACCCAAC |  |  |
| *embB* | Fw: embBF^b^ | CGACGCCGTGGTGATATTCG | 56 | 863 |
|  | Rev: embBR^b^ | CCACGCTGGGAATTCGCTTG |  |  |
| *gyrA* | Fw: gyrAF^b^ | ACCGGTTGACATCGAGCAGGAG | 59 | 592 |
|  | Rev: gyrAR^b^ | CTCTTCGTCGGCGTCGTGATTC |  |  |

^a^The oligonucleotide primers newly designed in the present study**;** ^b^The oligonucleotide primers based on previously reported studies (1, 2).

**FIGURE S1. Georaphicial distribution of study sample.** Strains under the study originated from seven major cities of Punjab province of Pakistan.

**REFERENCES**

1. Bakuła Z., Napiórkowska A., Augustynowicz-Kopeć E., Zwolska Z., Jagielski T.: Mutations in the *embB* gene and their association with ethambutol resistance in multidrug-resistant *Mycobacterium tuberculosis* clinical isolates from Poland; BioMed Research International. 2013. <http://dx.doi.org/10.1155/2013/167954>.
2. Bakuła Z., Napiórkowska A., Kamiński M., Augustynowicz-Kopeć E., Zwolska Z., Bielecki J., Jagielski T.: Second-line anti-tuberculosis drug resistance and its genetic determinants in multidrug-resistant *Mycobacterium tuberculosis* clinical isolates. Journal of Microbiology, Immunology and Infection. doi: 10.1016/j.jmii.2015.04.003.
